# TRAF3 enhances TCR signaling by regulating the inhibitors Csk and PTPN22

**DOI:** 10.1038/s41598-017-02280-4

**Published:** 2017-05-18

**Authors:** Alicia M. Wallis, Ellie C. Wallace, Bruce S. Hostager, Zuoan Yi, Jon C. D. Houtman, Gail A. Bishop

**Affiliations:** 1 Graduate Program in Immunology, Iowa City, IA 52242 USA; 2 Biomedical Engineering, Iowa City, IA 52242 USA; 3 Depts of Microbiology, Iowa City, IA 52242 USA; 4 Internal Medicine, Iowa City, IA 52242 USA; 5 The University of Iowa and VAMC, Iowa City, IA 52242 USA

## Abstract

The adaptor protein TNF receptor associated factor (TRAF) 3 is required for effective TCR signaling and normal T cell effector functions, and associates with the CD3/CD28 complex upon activation. To determine how TRAF3 promotes proximal TCR signaling, we studied TRAF3-deficient mouse and human T cells, which showed a marked reduction in activating phosphorylation of the TCR-associated kinase Lck. The impact of TRAF3 on this very early signaling event led to the hypothesis that TRAF3 restrains one or both of two known inhibitors of Lck, C-terminal Src kinase (Csk) and protein tyrosine phosphatase N22 (PTPN22). TRAF3 associated with Csk, promoting the dissociation of Csk from the plasma membrane. TRAF3 also associated with and regulated the TCR/CD28 induced localization of PTPN22. Loss of TRAF3 resulted in increased amounts of both Csk and PTPN22 in T cell membrane fractions and decreased association of PTPN22 with Csk. These findings identify a new role for T cell TRAF3 in promoting T cell activation, by regulating localization and functions of early TCR signaling inhibitors.

## Introduction

The adaptor protein TRAF3 regulates effector functions in both CD4^+^ and CD8^+^ T cells, enhancing TCR signaling without altering overall numbers of mature T cells^1^. In contrast to conventional T cells, invariant NKT (iNKT) cell numbers decline sharply in the absence of TRAF3, due to a deficiency in TCR-induced upregulation of the transcription factor T-bet during iNKT development^2, 3^. It is thus important to understand the molecular mechanisms by which TRAF3 regulates early TCR signaling.

TRAF3 associates with the TCR complex following co-ligation of CD3 and CD28; ligation of neither alone is sufficient for effective TRAF3 recruitment^1^. T cell-specific TRAF3 deficient mice (T-*traf3* ^−/−^) revealed that loss of TRAF3 in conventional T cells leads to reduced CD3/CD28-stimulated activation of the TCR signaling proteins Linker of Activated T cells (LAT), CD3_ζ_-associated protein of 70 kDa (ZAP70), extracellular-regulated kinase (ERK) and phospholipase C-γ1 (PLC-γ1)^1^. Activation events upstream of ZAP70 phosphorylation were previously unexplored.

Induction of the TCR signaling pathway involves equilibrium between phosphorylation and de-phosphorylation of Lck at activating (Tyr^394^) and inhibitory (Tyr^5^°^5^) sites^4^. Lck activity is negatively regulated by both Csk and PTPN22, with Csk being the most widely recognized Lck inhibitor^5^. Csk phosphorylates Tyr^505^ of Lck^5^, which results in a conformational change in Lck that reduces the phosphorylation of the activating Tyr^394^ site^6^. The phosphatase PTPN22 inhibits Lck activation after TCR induction by dephosphorylating Tyr^394^ on Lck^7, 8^. PTPN22-mediated dephosphorylation of Lck prevents abnormally-prolonged TCR activation^9^.

Csk and PTPN22 associate in the cytoplasmic fraction of T cells, and this association dissipates shortly after TCR activation. After 15 minutes of TCR stimulation, Csk and PTPN22 begin to re-associate^7^. Previous studies identified an inherited human coding variant (PTPN22-R620W), associated with an increased likelihood of autoimmunity and susceptibility to pathogens such as *Mycobacterium tuberculosis* ^9^. The association between Csk and PTPN22 is defective in PTPN22-R620W-expressing cells^7, 10, 11^. These observations led to the hypothesis that TCR-induced Csk and PTPN22 disassociation regulates T cell activation by inhibiting TCR-associated proteins such as Lck and the CD3 complex^9^.

Recent studies demonstrate that TRAF3 associates with PTPN22 in PBMC^12^ and B cells^13^, and that the autoimmunity-associated R620W variant diminishes the association with TRAF3^12^. We thus hypothesized that TRAF3 promotes Lck activation and downstream events of early TCR signaling by regulating two key Lck inhibitors, Csk and PTPN22. We addressed this hypothesis using primary splenic T cells from T-*traf3* ^−/−^ mice and their WT littermate controls (LMC), as well as a complementary model of human T cell lines and TRAF3-deficient subclones that we produced. In TRAF3-deficient T cells, activation of Lck, as determined by Tyr^394^ phosphorylation, was consistently reduced. This suggested that the overall reduction in TCR signaling and TCR-dependent downstream functions in these T cells is primarily a consequence of this initial decrease in Lck activation. In WT T cells, TRAF3 association with Csk was dynamic, with the TRAF3-Csk complex re-locating from the membrane to the cytoplasm upon CD3/CD28 stimulation. In TRAF3-deficient T cells, decreased Lck activation levels coincided with an increase in Csk at the plasma membrane.

TRAF3 also associated with PTPN22 in T cells, and regulated PTPN22 localization to the membrane after TCR stimulation. Interestingly, the absence of TRAF3 reduced, but did not eliminate, the Csk-PTPN22 association, suggesting that TRAF3 enhances the formation of a potential tri-partite complex. The present findings reveal a new mechanism by which TCR signaling is regulated by the multi-functional adapter protein TRAF3.

## Results

### The impact of TRAF3 upon early TCR signaling events

TRAF3-deficient T cells display a ~50% decrease in TCR-mediated activation of signaling proteins, evident as early as phosphorylation of the tyrosine kinase ZAP70^1^. To address our goal of understanding how TRAF3 enhances early TCR signaling, we examined whether more proximal signaling events were also affected by a deficiency of TRAF3. We thus analyzed these events in primary splenic T cells from T-*traf3* ^−/−^ mice and their WT littermates (littermate controls, LMC). As a complementary model, we utilized shRNA in HuT28.11 human T cells to make a stable TRAF3-deficient subclone (shTRAF3), and a control subclone with luciferase-specific shRNA (shLUC). The shTRAF3 cells exhibited a ~60% reduction in TRAF3 protein compared to shLUC cells (Suppl. Fig. S1). Neither T-*traf3* ^−/−^ cells nor transduced subclones of HuT28.11 showed significantly altered total cellular levels of proteins required for TCR activation (Suppl. Fig. S1).

**Figure 1:**
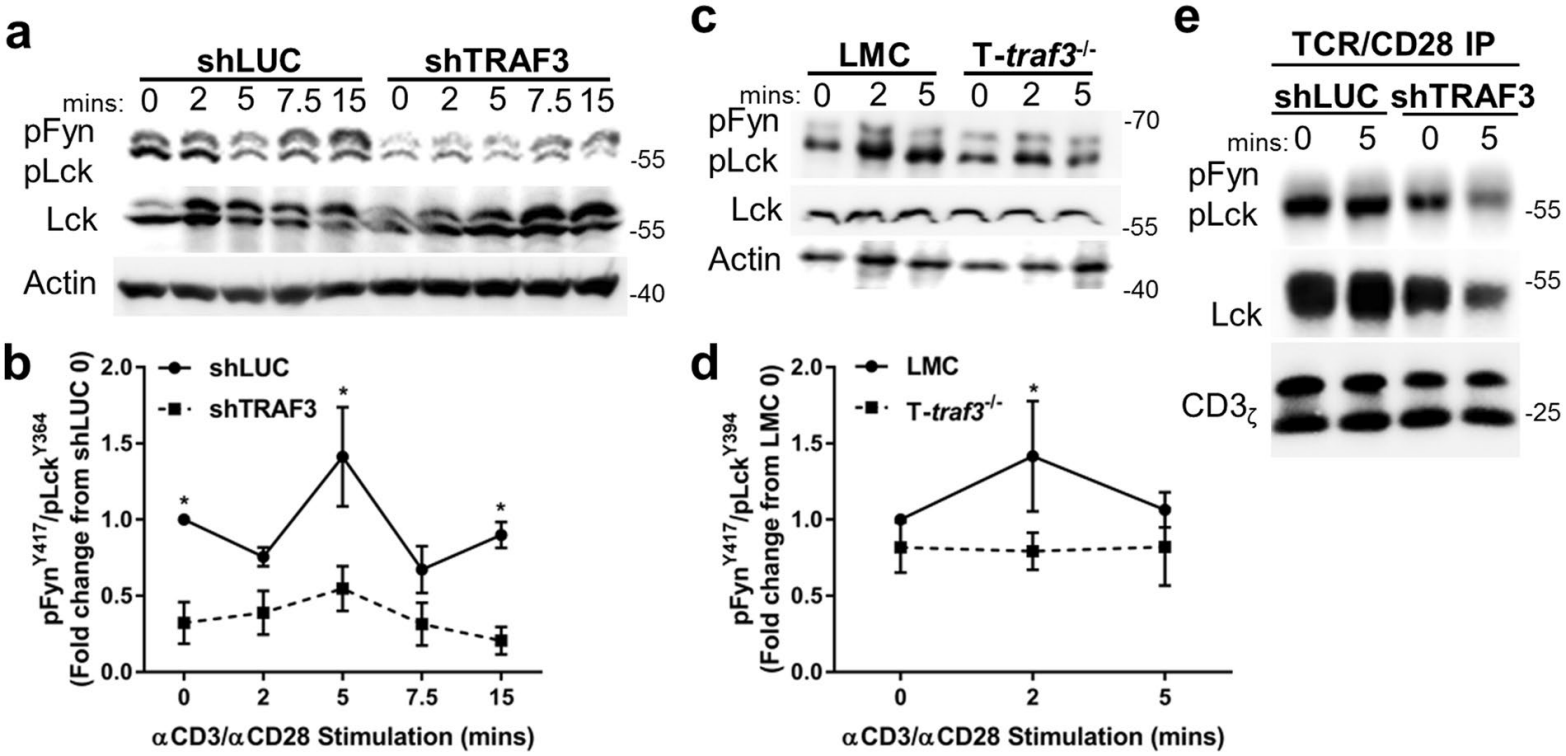
Requirement for TRAF3 in Src kinase activation by the TCR complex. T cells were stimulated via CD3/CD28 for indicated times. Whole cell lysates were prepared from HuT28.11 subclones shLUC or shTRAF3 (**a**), or primary mouse splenic T cells from LMC or T-*traf3* ^−/−^ mice (**d**) as described in Methods. Western blot analysis was performed to detect the indicated proteins. Expression levels of pFyn^Y417^/pLck^Y394^, detected by anti-pSrc^Y416^ Ab (**b** and **d**) were calculated as: (pLck/Actin)/(Lck/Actin) = pLck Relative expression level (REL). Fold change was then determined based upon the obtained expression levels, as follows. (pLck REL)/(pLck REL of control time point 0) = pLck Fold change. Data from at least 3 independent experiments were pooled and the mean values + SEM are shown. A 2-way ANOVA was performed to establish statistical significance; *P < 0.05. T-*traf3* ^−/−^ time point 7.5 is trending towards significance with p = 0.0597. (**e**) shLUC and shTRAF3 T cells were stimulated as described above. Immunoprecipitation of the TCR (CD3)/CD28 complex was performed on the whole cell lysates. The relative amount of pLck^Y394^ and total Lck association with the TCR/CD28 complex was analyzed by Western blot and blots were cropped to focus upon the specific proteins indicated. Full-length blots are presented in Suppl. Fig. S5. Data are representative of 3 individual experiments.

Using both these models, we examined one of the earliest measurable changes seen after engagement of the TCR complex, the activation of Src family kinases, particularly Lck. There was a basal decrease in pLck^Y394^ in human shTRAF3 cells that continued through 15 minutes of stimulation via CD3/CD28 (Fig. 1a,b). The most profound difference in relative levels of pLck^Y394^ occurred within 5 minutes of stimulation, so we focused upon early times post-stimulation in primary mouse T cells, to avoid unnecessary animal use. In the mouse model, TRAF3^−/−^ T cells displayed a similar decrease in activated pLck^Y394^ (Fig. 1c,d). Furthermore, both total and pLck^Y394^ association with the TCR/CD28 complex decreased in TRAF3-deficient human T cells (Fig. 1e). It is important to note that these data specifically evaluate Lck associated with the TCR/CD28 complex, rather than total membrane associated Lck, as a significant pool of membrane Lck will not be associated with the TCR/CD28 complex. Thus, these results do not suggest a reduction in levels of total membrane associated Lck. Additionally, utilizing CRISPR/Cas9-induced TRAF3 removal in a subclone of HuT28.11 T cells (crTRAF3^−/−^), phosphorylation of the CD3_ζ_ subunit of the TCR complex decreased in the absence of TRAF3 (Suppl. Fig. S2). Loss of TRAF3 in 2 different clones of crTRAF3^−/−^ T cell lines resulted in a reduction of Lck^Y394^ (Suppl. Fig. S2) and tyrosine phosphorylation levels of TCR signaling proteins after TCR/CD28 stimulation (Suppl. Fig. S3).

The reduction in both levels of pLck^Y394^ and the recruitment of Lck to the TCR/CD28 complex seen here extends previous work showing reduced phosphorylation of downstream TCR signaling proteins in the absence of TRAF3^1^, and reveals that the defect in TCR signaling in TRAF3-deficient T cells occurs very early in the TCR signaling cascade.

### TRAF3 association with Csk in T cells

**Figure 2:**
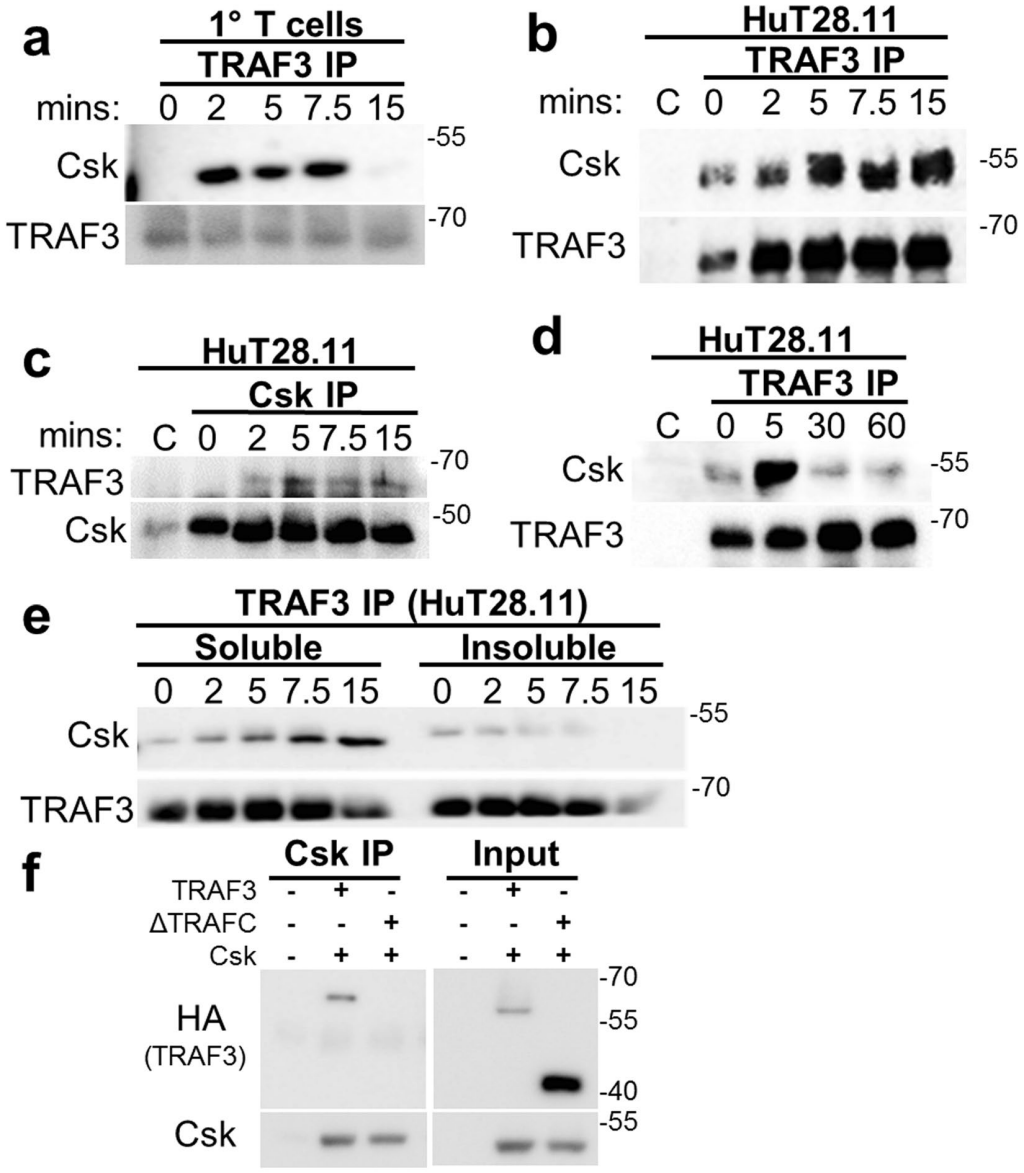
TRAF3 association with Csk in T cells. (**a**–**d**) T cells were stimulated via CD3/CD28 for the indicated times. Fab fragments targeting the stimulatory antibodies were added to prevent association of the stimulatory antibodies with the magnetic protein G beads. Samples were also precleared to remove any unbound stimulatory antibody. TRAF3 immunoprecipitation was then performed as described in Methods from whole cell lysates of WT primary mouse splenic T cells (**a**) or Hut28.11 T cells (**b** and **d**). (**c**) Immunoprecipitation of Csk from Hut28.11 whole cell lysates. **(e)** Cellular fractionation was performed as in Methods on HuT 28.11 T cells. TRAF3 was immunoprecipitated from soluble and insoluble cell lysate fractions, isolated as in Methods. (**a**–**e**) Analysis by Western blot to detect the indicated proteins. C = Control samples, in which cells were stimulated for 5′ and no immunoprecipitation Ab was added, to detect any residual stimulatory Ab association with the protein G beads. (**f**) HEK 293T cells transfected with TRAF3 and Csk constructs (depicted in Fig. 4a) were lysed, and Csk was immunoprecipitated. Total input prior to immunoprecipitation is shown at right. Full-length blots are presented in Suppl. Fig. S5. Data shown are representative of 3–6 independent experiments.

The Lck inhibitor Csk contains both SH2 and SH3 domains. As TRAF3 contains several potential SH2 and SH3 binding sites, we predicted that TRAF3 associates with Csk in T cells. This prediction was confirmed in immunoprecipitated TRAF3 from mouse splenic T cells, following CD3/CD28 stimulation. This interaction remained robust for 5–7.5 minutes post-stimulation, returning to near-unstimulated levels after 15 minutes (Fig. 2a). TRAF3 also associated with Csk in HuT28.11 human T cells (Fig. 2b). Consistent with the nature of transformed cells, some association was seen even in unstimulated HuT28.11 cells. However, as in the primary mouse T cells, this association increased upon CD3/CD28 activation. Reciprocally, immunoprecipitation of Csk in HuT28.11 T cells confirmed the inducible association of Csk with TRAF3 (Fig. 2c).

To further investigate the TRAF3:Csk association, extended CD3/CD28 activation was performed in human T cells, revealing a decline in association by 30 minutes (Fig. 2d). Due to the disassociation of Csk from the membrane after TCR stimulation, and the different cellular compartments in which Csk can reside^5^, we performed cell fractionation to determine where the association between TRAF3 and Csk occurred. Results indicated a dynamic association between TRAF3 and Csk in distinct cell sub-compartments. Upon CD3/CD28 stimulation, levels of associated Csk:TRAF3 decreased in the plasma membrane fraction, while increasing in the cytoplasmic fraction (Fig. 2e). Association with Csk prior to engagement of the TCR complex involves the membrane pool of TRAF3. Thus, the Csk-TRAF3 association seen in resting T cells likely involves the membrane pool, while the increased association following stimulation can involve both this pool, as well as the newly-recruited TRAF3 from the cytoplasm.

To determine if the interaction of Csk and TRAF3 requires the TRAFC domain, which typically participates in protein-protein interactions involving TRAF3^14^, HEK 293T cells were transfected with plasmids containing full length Csk and WT TRAF3, or TRAF3 lacking this domain (ΔTRAFC) (Fig. 2f). Co-immunoprecipitation confirmed the TRAF3:Csk association, and indicated that the TRAFC domain of TRAF3 was required for this association (Fig. 2f). Together, these results are consistent with the hypothesis that T cell TRAF3 associates with Csk at the plasma membrane and facilitates translocation of Csk to the cytoplasm upon TCR/CD28 stimulation.

### Csk regulation by TRAF3

**Figure 3:**
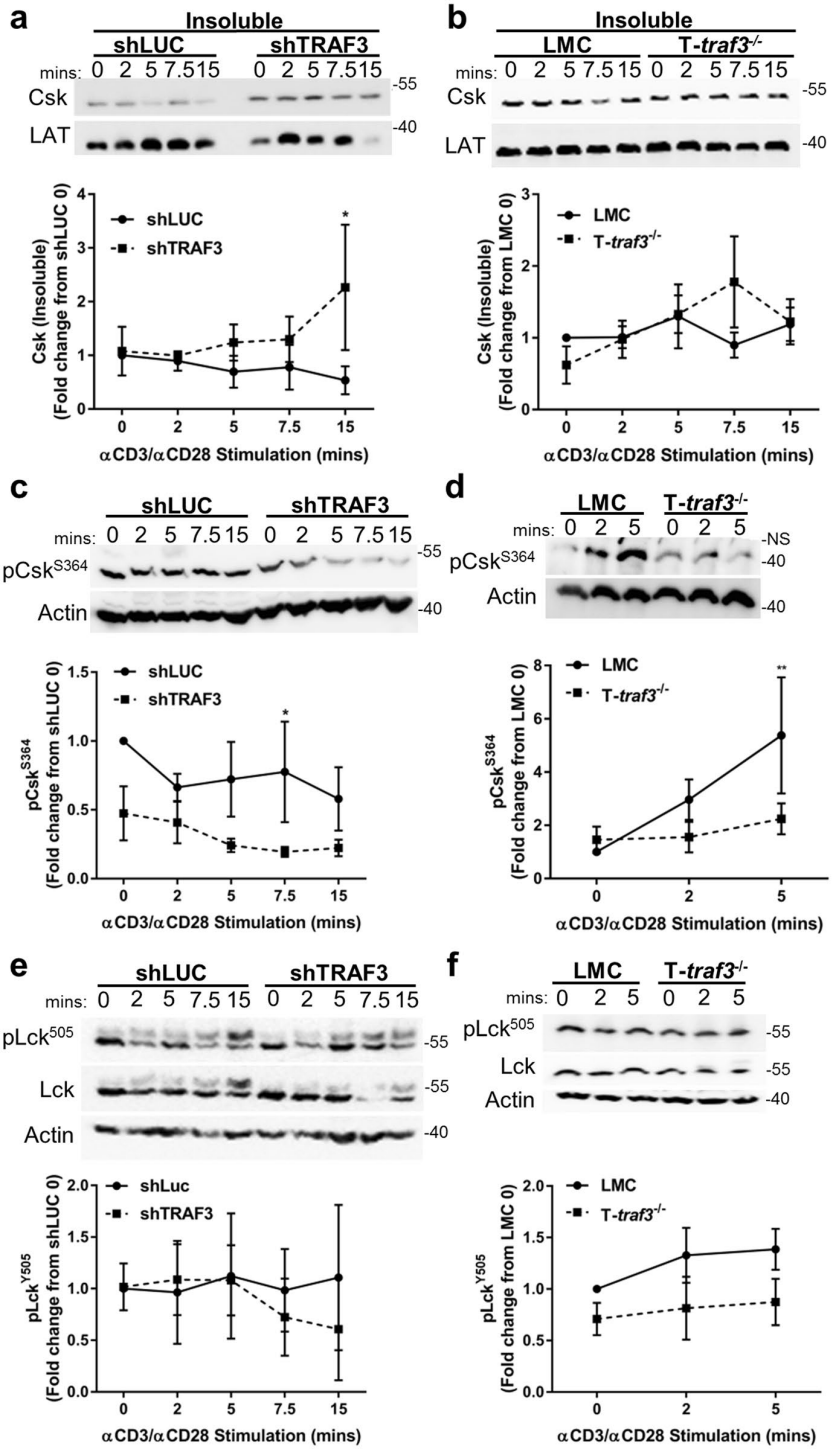
Role of TRAF3 in the regulation and localization of Csk. T cells were stimulated with αCD3/αCD28 Abs for the indicated times and a cellular fractionation was performed, as described in Methods, on lysates from shLUC and shTRAF3 cells (**a**) or isolated primary mouse splenic T cells from LMC and T-*traf3* ^−/−^ mice (**b**). Western blot analysis of the insoluble (membrane) fractions was performed to detect the designated proteins, with actin or LAT serving as loading controls (Top panel). Expression levels of Csk were first normalized to actin and the subsequent fold change was calculated from the control 0 time point (bottom panel). Data from T-*traf3* ^−/−^ cells stimulated for 7.5 minutes is trending towards significance with p = 0.0597. Western blot analysis was performed on whole cell lysates from human T cell lines (**c**) or primary mouse splenic T cells (**d**) and blotted for pCsk^S364^ (Top panel). Expression levels of pCsk^S364^ were first normalized to actin and subsequent fold change was calculated from the control 0 time point (bottom panel). Western blot analysis was performed on whole cell lysates to detect the expression levels of pLck^Y505^ (**e** and **f**, top panel). Western blots were cropped to focus upon specific proteins indicated. Expression levels of pLck^Y505^ were first normalized to actin and subsequent fold change was calculated from the control 0 time point. Differences between TRAF3-deficient and TRAF3-sufficient cells did not reach statistical significance. Data from at least 3 independent experiments were pooled and the mean values + SEM are shown. A 2-way ANOVA was performed to determine statistical significance in all 6 panels; *P < 0.05, **P < 0.01. NS = Nonspecific band. Full-length blots are presented in Suppl. Fig. S5.

Results discussed above suggest that TRAF3 recruited to the TCR complex affects relative Csk levels in the plasma membrane fraction of T cells. Following CD3/CD28 stimulation, shTRAF3 T cells displayed an increase in Csk in the membrane fraction, compared to control T cells (Fig. 3a). TRAF3^−/−^ mouse primary T cells also had an increase in membrane-associated Csk at the peak association time revealed in Fig. 2 (7.5 minutes), whereas membrane Csk levels in LMC T cells showed a marked decrease at this time (Fig. 3b).

Phosphorylation of Csk at S^364^ inhibits its catalytic activity^15, 16^, so we next examined whether T cell TRAF3 influences this mechanism of Csk regulation. In both shTRAF3 and TRAF3^−/−^ T cells, pCsk^S364^ levels were decreased compared to control T cells (Fig. 3c,d). These findings suggest that more Csk is in the activated state in TRAF3-deficient T cells.

The increase in Csk at the membrane and the reduction in levels of pCsk^S364^ predicted a correlative increase in pLck^Y505^ phosphorylation in TRAF3-deficient T cells. However, there was no detectable increase in the level of pLck^Y505^ in either shTRAF3 or TRAF3^−/−^ T cells. The modest apparent decrease in pLck^Y505^ is not statistically significant (Fig. 3e,f). Several possibilities could account for these results. Technical limitations of Western blotting may fail to reveal transient changes. Alternatively, the reduction in TCR signaling seen in the absence of TRAF3 is not solely dependent on Csk. This increased our interest in further investigating the interactions between TRAF3 and Csk’s binding partner, the phosphatase PTPN22.

### TRAF3 regulation of PTPN22-Csk association

**Figure 4:**
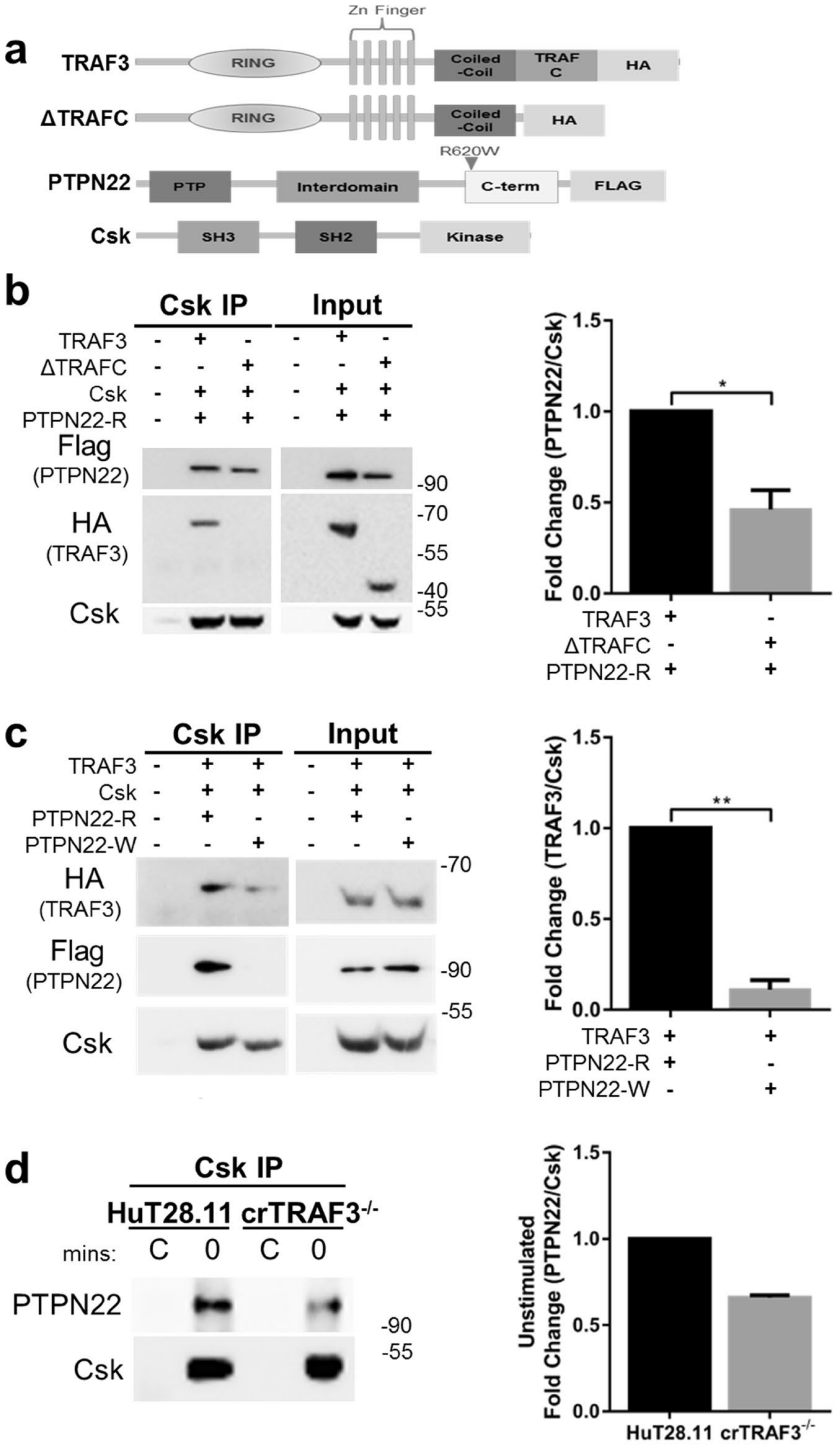
TRAF3, PTPN22 and Csk association. (**a**) TRAF3, PTPN22 and Csk constructs used in HEK 293T cell transfections. (**b** and **d**) Immunoprecipitation of Csk from transfected HEK 293T cell lysates is shown at left, with total cell lysate input prior to immunoprecipitation shown at right. Western blot analysis was performed for the specified proteins. Quantification of bands was performed by dividing the relative expression of PTPN22 by Csk (**c**) or TRAF3 by Csk (**e**). The obtained values for the cells transfected with the mutant plasmids were then normalized to the values obtained for cells transfected with the control wild type plasmids. (**d**) From unstimulated crTRAF3^−/−^ (clone 45) T cell whole cell lysates, an immunoprecipitation for Csk was performed as described previously. Western blots were cropped to focus upon specific proteins indicated. Full-length blots are presented in Suppl. Fig. S5. C = Control samples, cells were unstimulated and no immunoprecipitation Ab was added, to detect any nonspecific binding to the protein G beads. Error bars indicate SEM of three experiments. A Wilcoxon matched-pairs signed rank test was performed to determine statistical significance in **b** and **c**; *P < 0.05, **P < 0.01. Data are representative of 3–8 independent experiments.

Csk associates with PTPN22 in the cytoplasm of resting human T cells. Upon TCR stimulation, PTPN22 is released from Csk to translocate to the membrane, where it dephosphorylates Lck^Y394^ ^7^. Interestingly, the variant PTPN22-R620W has markedly decreased association with Csk^10^, and the same variant displays decreased TRAF3-PTPN22 association^12^. These reports, together with the findings above demonstrating T cell Csk-TRAF3 association, led us to ask whether TRAF3 competes with Csk for association with PTPN22 at amino acid 620. We also wished to know whether all three proteins could form a complex together, and whether each individual protein is required for this complex to form. HEK 293T cells were transfected with constructs encoding full length or mutant TRAF3 and PTPN22, together with full length Csk (Fig. 4a). In cells expressing WT TRAF3, PTPN22 and Csk, both TRAF3 and PTPN22 associated with Csk (Fig. 4b). The association between Csk and PTPN22 was reduced in the presence of the ΔTRAFC mutant, which does not associate with Csk (Fig. 4b). There was also a modest reduction in the total level of PTPN22 in the ΔTRAFC mutant transfected cells. These data indicate that optimal interactions between Csk and PTPN22 require WT TRAF3 (Fig. 4b). Association between Csk and TRAF3 was also reduced in cells co-transfected with PTPN22-R620W, compared to WT PTPN22 (Fig. 4c). Furthermore, utilizing the crTRAF3^−/−^ T cell line, we found association between Csk and PTPN22 decreased in the absence of TRAF3 in unstimulated cells (Fig. 4d) with a similar reduction in association in WT cells upon TCR/CD28 stimulation (Suppl. Fig. S4). Together these data indicate that Csk, TRAF3 and PTPN22 can potentially form a complex together, and both TRAF3 and PTPN22 are required for maximum association of the reciprocal protein with Csk. That all three proteins are not absolutely required for the association supports the model that there are both TRAF3-dependent effects on Csk or PTPN22, but there are also TRAF3-independent roles for Csk and PTPN22 complexed together.

### TRAF3 regulation of PTPN22 localization

**Figure 5:**
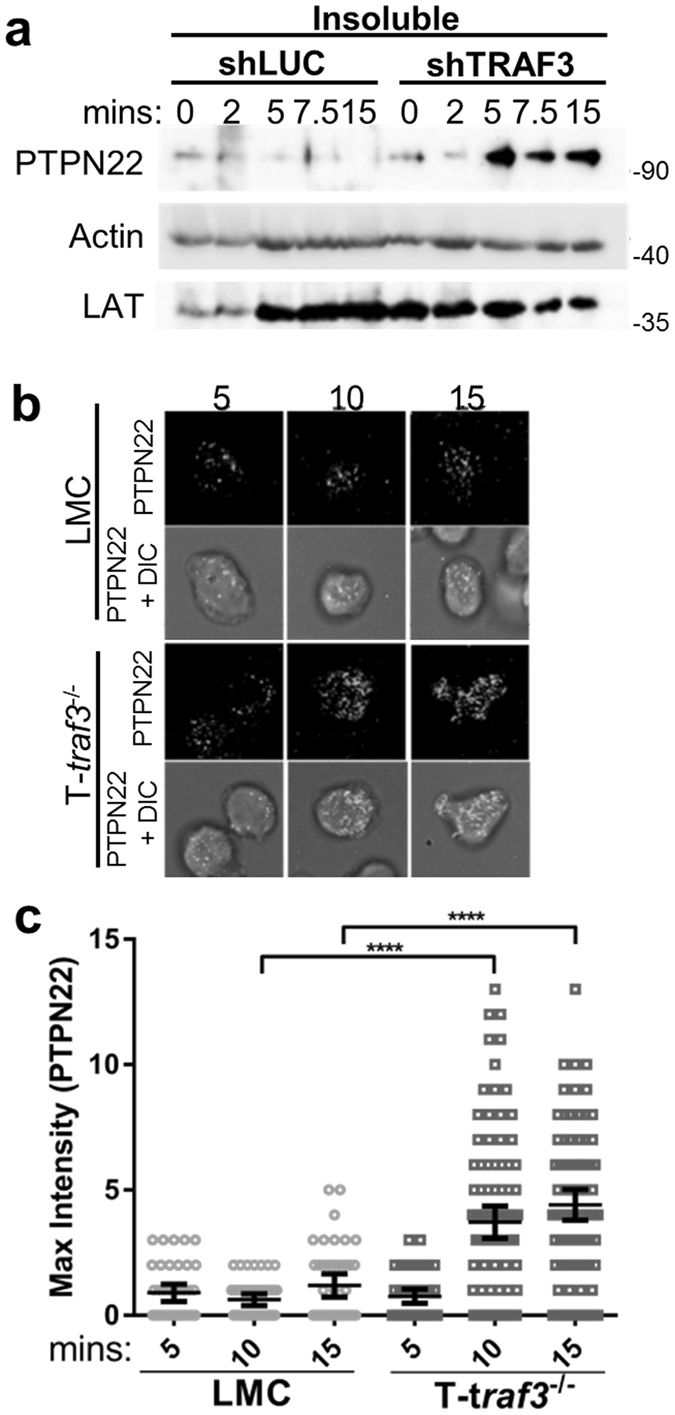
TRAF3 regulation of PTPN22 localization and association with membrane Csk. T cells were stimulated for indicated times via CD3/CD28 (**a** and **b**). Cellular fractionation was performed on shLUC and shTRAF3 T cells to obtain the insoluble (membrane) fractions. (**a**) Total PTPN22, Lck and actin expression in the insoluble cell fraction was determined by Western analysis using the respective Abs. Western blots were cropped to focus upon the designated proteins. Full-length blots are presented in Suppl. Fig. S5. (**b** and **c**) Primary mouse splenic T cells isolated from LMC and T-*traf3* ^−/−^ mice were permeabilized and stained with an anti-PTPN22 Ab followed by a secondary Ab for Alexa-488, then analyzed by Total Internal Reflection Fluorescence (TIRF) microscopy. (**b**) Representative TIRF images and (**c**) quantification of each cell for maximum intensity of PTPN22 fluorescence was determined as in Methods (n = 50 cells per time point). A 1-way ANOVA was performed to establish statistical significance, *P < 0.0001. Data are representative of 3 (**a**) or 2 (**b** and **c**) independent experiments.

Because the reduction in pLck^Y394^ in TRAF3-deficient T cells was not demonstrably correlated with an increase in pLck^Y505^ (Fig. 3), and Fig. 4 reveals that PTPN22 can associate with both TRAF3 and Csk, we hypothesized that TRAF3 regulates PTPN22-induced dephosphorylation of Lck^Y394^ ^7, 8^. First, we queried whether the relative amount of PTPN22 at the T cell plasma membrane is regulated by TRAF3. In CD3/CD28-stimulated TRAF3-deficient human T cells, more PTPN22 was observed in the insoluble membrane fraction of cell lysates, compared to control T cells (Fig. 5a).

To further address the potential role of TRAF3 in PTPN22 trafficking within intact T cells, we examined PTPN22 localization in mouse splenic T cells stimulated with plate bound αCD3/αCD28 Abs, using Total Internal Reflection Fluorescence (TIRF) microscopy (Fig. 5b). TIRF microscopy permits visualization of proteins within 100–150 nM of a coverslip upon which cells are stimulated. This allowed us to examine PTPN22 localized to the plasma membrane, without the distraction of background cytoplasmic fluorescence. We observed an increase in PTPN22 clustering at the membrane in TRAF3^−/−^ compared to LMC T cells (Fig. 5c). Taken together, these data indicate that TRAF3 restrains the amount of PTPN22 associated with the membrane after CD3/CD28 stimulation. Results thus indicate that TRAF3 regulates relative levels of pLck^Y394^ and TCR activation by controlling the amount of PTPN22 migrating to the membrane.

## Discussion

It is becoming increasingly apparent that there are multiple roles for TRAF3 in T cell biology. While there are normal numbers of conventional CD4^+^ and CD8^+^ T cells in the T-*traf3* ^−/−^ mouse, these mice display markedly defective *in vivo* responses to immunization, including providing effective help to induce a B cell response, and to infection with *L. monocytogenes*. Additionally, TRAF3^−/−^ primary T cells have striking defects in IL-2, IL-4, TNFα and IFNγ cytokine production, in response to CD3/CD28 stimulation. These findings led to the discovery that TRAF3 associates with the TCR complex upon CD3/CD28 stimulation, and TCR signaling is significantly impaired in the absence of TRAF3^1^. While this reduced TCR function is sufficient to allow the development of conventional T cells, it results in a major reduction in iNKT cells^2^, which may also contribute to defective *in vivo* immune responses. Retroviral transduction of TRAF3 into T-*traf3* ^−/−^ mice rescued the developmental defect in iNKT cells, by increasing TCR-induced T-bet expression^2^.

It was thus a high priority to understand how TRAF3 enhances TCR signaling, the focus of the present study. Loss of TRAF3 in our T-*traf3* ^−/−^ mouse model results in reduction in activation of several TCR signaling proteins, including ZAP70, LAT, ERK and PLCγ1^1^. We demonstrate here that TRAF3 enhanced the earliest detectable TCR signaling event, the activation of Src kinases. In the absence of TRAF3, less Lck is associated with the TCR/CD28 complex. This suggests that upon CD3/CD28 stimulation, less membrane associated Lck in TRAF3-deficient T cells is recruited to the TCR/CD28 complex, preventing Lck from phosphorylating the CD3 subunits of the TCR. This does not suggest that the reduction in Lck association with the TCR/CD28 complex is a result of decreased total membrane levels of Lck; we found no data to support this.

**Figure 6:**
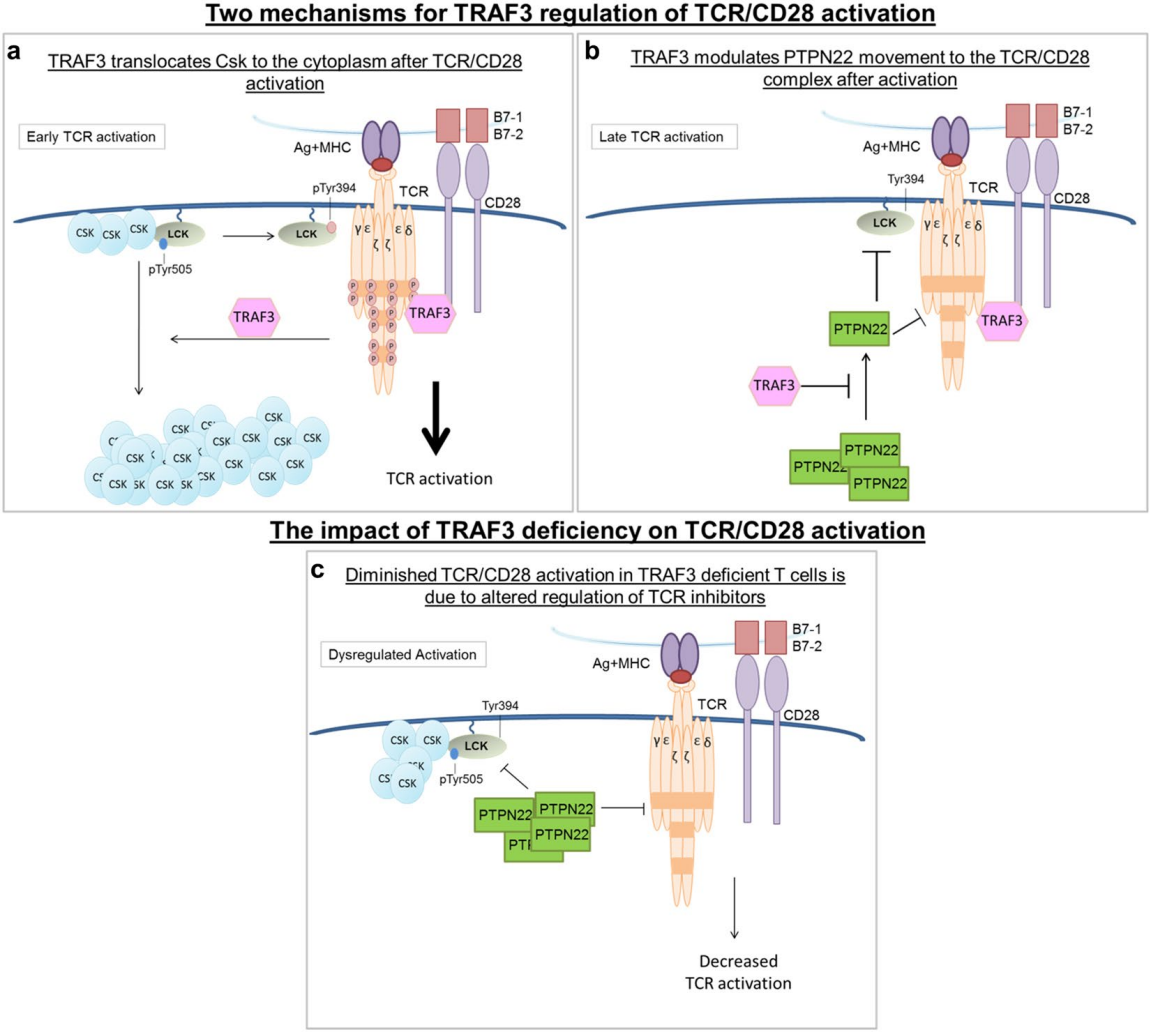
TRAF3 regulation of Csk and PTPN22 in TCR/CD28 signaling. TRAF3 regulates TCR signaling via at least 2 distinct mechanisms. During early TCR activation, TRAF3 associates with Csk to translocate Csk away from the plasma membrane to the cytoplasm (**a**). This allows the recruitment of activated Lck to the TCR complex where Lck phosphorylates the CD3 subunits. A second mechanism of TRAF3 regulation of TCR/CD28 signaling is by controlling the localization of cytosolic PTPN22 to the plasma membrane. To inhibit prolonged TCR/CD28 activation, TRAF3 regulates the amount and time at which PTPN22 is released to the membrane from the cytoplasm (**b**). Once at the membrane, PTPN22 dephosphorylates Lck and the CD3 subunits of the TCR complex to inhibit TCR/CD28 signaling. In the absence of TRAF3 both mechanisms of TCR/CD28 inhibition are enhanced. The loss of TRAF3 results in more Csk at the membrane and more PTPN22 recruited to the membrane earlier in TCR/CD28 activation (**c**).

TRAF3 enhances TCR signaling by regulating the localization of two inhibitors of TCR and Lck signaling, the kinase Csk and the phosphatase PTPN22. The first mechanism by which TRAF3 regulates TCR/CD28 signaling occurs upon CD3/CD28 activation, when TRAF3 sequesters Csk away from the plasma membrane (Fig. 6a). Removal of Csk from the membrane allows more Lck to become activated and subsequently phosphorylate the TCR complex. Because the current study indicates that TRAF3 regulates Csk movement, an area for further research is to determine the mechanism that leads to TRAF3:Csk association. There are two pools of TRAF3 that have the potential to associate with and subsequently regulate Csk: membrane-localized TRAF3 and TRAF3 recruited to the TCR complex from the cytoplasm in activated T cells. It is of particular interest to determine if TRAF3 in different cellular compartments associates with and regulates Csk differently.

Several minutes after the activation of TCR/CD28, TRAF3 also regulates the translocation of PTPN22 to the membrane (Fig. 6b). Following this translocation, signaling proteins such as Lck and the CD3 subunits of the TCR complex are dephosphorylated and inhibited. This allows TRAF3 to regulate the duration and intensity of TCR/CD28 signaling. In the absence of TRAF3, there are increased levels of both Csk and PTPN22 at the membrane. In TRAF3 deficient T cells, Csk is not removed from the plasma membrane upon stimulation, while PTPN22 protein levels not only significantly increase at the membrane, but the increase is also seen earlier after stimulation (Fig. 6c).

Approximately 80% of T cell Csk is found in the cytoplasm, while ~20% is located at the plasma membrane^5^. Thus, even modest alterations in the plasma membrane levels of Csk have the potential to significantly alter Lck activation, and result in substantial impact upon early TCR signaling. In a study by Manz *et al*., low doses of a Csk small molecule inhibitor increase pLck^Y394^ levels, while a reciprocal decrease in the level of pLck^Y505^ does not occur^17^, similar to the effects of TRAF3 shown here. Importantly, an increase in Lck activation of less than 50% is sufficient to alter TCR signaling^17^. Focal Adhesion Kinase (FAK), which recruits Csk to the membrane, fine-tunes TCR signaling, similar to the impact of TRAF3, by regulating the levels of Csk at the plasma membrane^18^. In FAK-deficient T cells, slight decreases in Csk levels are sufficient to alter Lck activation and thus lead to increased TCR signaling^18^. TRAF3 increasingly associated with Csk in the cytoplasm, peaking at 15 minutes, with a reciprocal decrease in association at the membrane. Because the association between Csk and FAK occurs earlier than 15 minutes, this suggests TRAF3 is not associating with FAK to return Csk to the membrane^18^. These data suggest that subtle alterations in Csk and Lck can have substantial effects on TCR signaling. The manner in which Csk is removed from the membrane upon CD3/CD28 stimulation has been a knowledge gap in the field of TCR signaling for some time; the present results indicate that TRAF3 can serve in this role.

Previous research also identified an association between Csk and PTPN22 in T cells, and between TRAF3 and PTPN22 in other cell types, as discussed earlier. Our current results suggest that all three signaling proteins can potentially associate to form a complex, and that both TRAF3 and PTPN22 are required for optimal association of the three proteins. TRAF3 also regulated the localization of PTPN22 to the cellular membrane after TCR stimulation. In the resting T cell, PTPN22 is located in the cytoplasm, where it associates with Csk until stimulation. These data suggest that the cytoplasmic pool of TRAF3 regulates the release of PTPN22 to the membrane and the association of PTPN22 with Csk. This indicates that TRAF3 impacts the dynamics of termination of TCR signaling pathways after activation. In the absence of TRAF3, increased levels of PTPN22 were observed at the membrane, pointing to TRAF3 as a rheostat to fine-tune TCR signaling by regulating the localization of key inhibitory molecules.

Findings presented here indicate that the role of the potential TRAF3:Csk:PTPN22 tri-molecular complex in TCR signaling is worthy of additional future investigation. In myeloid cells, PTPN22 induces the lysine 63-linked ubiquitination of TRAF3^12^. Thus, PTPN22 may form a complex with TRAF3 to regulate TRAF3 through its ubiquitination in this cell type. Another interesting question is whether TRAF3 is required for PTPN22 to regulate the function of Csk. While we examined the phosphorylation of Csk on S364, previous publications have also identified potential tyrosine phosphorylation sites on Csk, Y184 and Y304^19^. PTPN22 can potentially dephosphorylate both sites to regulate Csk activity. Unfortunately, there are no specific antibodies at this time that detect the tyrosine phosphorylation of Csk. How post-translational modifications of TRAF3, Csk, and PTPN22 regulate their interactions and T cell-specific functions is an intriguing topic for future investigation. Finally, we identified an association between Csk and TRAF3 or PTPN22 independent of the reciprocal protein. This suggests Csk can associate with TRAF3 and PTPN22 independently and thus each association may have different functions in TCR signaling.

We show here that TRAF3 enhances TCR signaling by fine-tuning the localization of TCR signaling inhibitors. A future question of interest is how TRAF3 associates with the TCR/CD28 complex upon CD3/CD28 stimulation. TRAF3 is predominately found in the cytoplasm, though there is significant expression of TRAF3 in the membrane of unstimulated cells^20^. As discussed earlier, TRAF3 is recruited to the TCR/CD28 complex only after stimulation of both CD3 and CD28, and this could include both recruitment of membrane-localized TRAF3, as well as recruitment from the cytoplasmic pool. One possibility is that TRAF3 associates with the TCR or CD28 receptor directly. Association of TRAF3 with the TCR/CD28 complex is enhanced by the clustering of the TCR and CD28 receptors upon TCR activation. Stimulation through either CD3 or CD28 alone reduces the clustering typically seen with full CD3/CD28 activation, which would result in decreased TRAF3 association. Alternatively, TRAF3 could associate with a TCR signaling adaptor protein, such as LAT. In this case, stimulation-induced TCR clustering would result in the adapter protein bringing TRAF3 to the TCR/CD28 complex, resulting in an indirect association between the TCR or CD28 receptors and TRAF3. We anticipate that additional TCR-related roles for TRAF3 may be revealed in the future.

## Materials and Methods

### Mice

*Traf3* ^flox/flox^ mice, backcrossed >10 generations to C57Bl/6 mice^21^, and crossed to *Cd4*-Cre mice^22^, created the T-*traf3* ^−/−^ strain used here, together with their littermate controls (LMC). Adult mice (2–4 months) were used for all experiments. Mice were housed in specific pathogen-free conditions and used in accordance with NIH guidelines, under a protocol approved by the Animal Care and Use Committee, University of Iowa.

### Cell lines

A subclone of the human CD4^+^ T cell line HuT transfected to stably express CD28 (HuT28.11) was the gift of Dr. Arthur Weiss, University of California, San Francisco^23^. HuT28.11 cells were cultured in RPMI 1640 medium supplemented with 100 U/ml penicillin, 100 U/ml streptomycin, 2 mM L-glutamine, 10 μM β-mercaptoethanol, and 10% fetal calf serum (FCS) (BCM10). Subclones of HuT28.11, shLUC, shTRAF3 and crTRAF3^−/−^, are described below; culture included 2 mg/ml G418.

### Viral RNA constructs, cell culture and transduction

Production of HuT28.11 subclones stably expressing the small hairpin (sh) RNA constructs shLUC (vector control) and shTRAF3 was performed as indicated^24, 25^, with minor modifications as follows. Using the *hTRAF3* sequence as a template, shRNAs targeting *TRAF3* were obtained from the algorithm of Dr. Ravi Sachidanandam (http://katahdin.cshl.edu). The following sequences were used for production of shTRAF3 (TRAF3–8 sense 5′ GAACCTACCGGTCCGTGTGTCCCTGCTCATAAAGTAGTGAAGCCACAG 3′ TRAF3–8 anti-sense 5′ GTTCCGAATTCAAAAAATCGTGTGTCCCTGCTCATAAAGTACATCTGTGGCTTC3′; TRAF3–14 sense 5′GAACCTACCGGTAACTGGTTATCACTTGTGATAGTAGTGAAGCCACAG 3′ TRAF3–14 anti-sense 5′GTTCCGAATTCAAAAAACACTGGTTATCACTTGTGATAGTACATCTGTGGCTTC 3′). Both shTRAF3–8 and shTRAF3–14 were used together to produce the most effective inhibition of TRAF3 expression.

To make shRNA-containing virus, HEK 293T cells were transfected using lipofectamine 2000, according to the manufacturer’s instructions. Each transfection included 5 μg of each shRNA plasmid (pLKO.1 shTRAF3–8 and −14), with viral packaging vectors VSV-G (4 μg), and Pax2 (10 μg). This mixture was incubated at 37 °C for 6–8 h, washed, and cultured with 25 ml fresh DMEM10 supplemented with 100 U/ml penicillin, 100 U/ml streptomycin, 2 mM L-glutamine, 10 mM HEPES, 1 x MEM NEAA, and 10% FCS. Culture supernatant containing recombinant virus was collected at 24 and 48 h and isolated as in ref. 26. Virus was resuspended in 1.5 ml BCM10. HuT28.11 T cells (3–5 × 10^5^) were resuspended in 1.5 ml of virus-containing supernatant, with 8 μg/ml hexadimethrine bromide (Polybrene). Cells were cultured for 1 week, after which shRNA-expressing cells were selected with 1 μg/ml puromycin.

### Production of crTRAF3^−/−^ subclone

Guide RNA/Cas9 vector constructs for disruption of the *TRAF3* gene were prepared as described^27^, using the CRISPR design tool (crispr.mit.edu) maintained by Dr. Feng Zhang (MIT, Cambridge, MA). Two constructs were prepared, one targeted to intron 1 upstream of the ATG, and a second to exon 5. The double-stranded synthetic oligonucleotides for intron 1 were: 5′ CACCGCCATCATATCCTCTCATGCA 3′, and 5′ AAACTGCATGAGAGGATATGATGGC 3′ (IDT). The exon 5 oligonucleotide pairs were 5′ CACCGGTTCCGATGATCGCGCTGC 3′ and 5′ AAACGCAGCGCGATCATCGGAACC 3′. Pairs were annealed and phosphorylated as per^27^. pX330 (Addgene ID 42230) was cut with BbsI and treated with calf intestinal phosphatase, then purified (QIAquick PCR purification column, Qiagen). Phosphorylated double-stranded oligonucleotides were ligated into the cut vector and ligated DNA used to transform competent *E. coli*. Plasmid DNA was sequenced to verify proper insertion. 2.5 × 10^6^ HuT28.11 cells were resuspended in 400 ul Optimem with 2.5 ug of each of the two guide RNA/Cas9 vectors, 0.5 ug pEGFP-C1 (Clontech), and 5 ug double-stranded filler DNA oligonucleotides (random sequence^28^). The cell suspension was electroporated in 4 mm cuvettes, 225 V for 30 ms (BTX square wave electroporator). After a 10′ rest at 37 °C, cells were resuspended in 10 ml BCM10 and cultured for 5d. GFP-expressing cells were sorted at 1 cell/well into 96-well plates. Clones were screened by PCR of genomic DNA using the following primers: 5′ CTGAAAGACAGCAGGTCTCAGGCAC 3′, and 5′ GAATGTATCATATAGGAATTGAGTGG 3′ (Int-5R3). A PCR product of ~100 bp indicated the desired deletion. DNA samples exhibiting this product were retested with primers specific for sequences within the deleted region (5′ GGTTTCATTGCATAGAGATTAGAATC 3′, and Int-5R3 (above)). Clones testing negative for the 300 bp intact gene product were screened by Western blot to confirm disruption of TRAF3 protein expression.

### Immunoprecipitation

Primary mouse splenic T cells were isolated using a Pan T cell negative purification kit (StemCell Technologies). 30 × 10^6^ primary T or HuT28.11 T cells or subclones were used/time point. Cells were washed with serum- free RPMI 1640 medium and stimulated at 37 °C with anti-CD3 and anti-CD28 stimulatory mAbs at 10 μg/ml. Cell lysis was performed as in^24^ with slight modifications as follows. Cells were lysed using 500 μl of Brij 97 buffer (25 mM Tris pH 8.0, 150 mM NaCl, 1% Brij-97, 0.5% n-Octyl-β-d-glucopyranoside, 2 mM Na_3_VO_4_, EDTA free mini-complete protease inhibitor tablets, and 5 μg/ml DNase 1. AffiniPure F(ab’)^2^ Ab fragments (mouse T cells: rabbit anti-syrian hamster IgG) (human T cells: goat anti-mouse IgG), were added to each lysate (10 μl/IP) to inhibit stimulatory Ab association with protein G beads. Lysates were pre-cleared with protein G beads to remove nonspecific binding. Lysates were incubated ± immunoprecipitation Abs overnight on a rotator at 4 °C (The lane label C indicates the sample was stimulated for 5′ but no immunoprecipitation Ab was added). The following Abs were used for immunoprecipitation: 2 μg of rabbit anti-TRAF3 or 5 μl of mouse anti-Csk mAb. The next day protein G beads were added for 1 h, rotating at 4 °C. Using a magnet, beads were washed 3X with 25 mM Tris pH 8.0, 150 mM NaCl, 0.5% SDS. To elute immunoprecipitated proteins, 2X sample buffer was added to the beads and subsequently boiled at 95 °C for 5′. Samples were separated by SDS-PAGE and analyzed via Western blotting. For TCR complex (CD3 + CD28) immunoprecipitation, the above protocol was used with the following alterations. Rabbit anti-mouse IgG (3.5 ul/IP) was added instead of the F(ab’)^2^ Ab fragments to crosslink the CD3/CD28 antibodies.

### Cell fractionation

T cells were washed and stimulated as above. After activation, cells were resuspended in a solution of 10 mM HEPES [pH 7.4], 5 mM MgCl_2_, 2 mM Na_3_VO_4_, EDTA-free mini-complete protease inhibitor tablets, and placed on ice for 15′. Cells were sheared by passage 5X through a l ml syringe with a 27 g needle. The lysate was centrifuged at 200 × g for 10′. The supernatant was recovered and subjected to ultra-centrifugation (Beckman) at 90,000 × g for 3 h. The supernatant was saved and the pellet resuspended in membrane solution (20 mM Tris [pH7.4], 150 mM NaCl, 1% NP-40, 2 mM Na_3_VO_4_, EDTA free mini-complete protease inhibitor tablets). Immunoprecipitation from the cytoplasmic and membrane fractions was performed as described above using 5 μl anti-PTPN22 mAb or 2 μg rabbit anti-TRAF3 Ab.

### HEK 293T cell transfection

HEK 293T cells (1 × 10^6^/well of a 6-well plate) were grown overnight in DMEM. Prior to transfection, DMEM was replaced with Optimem-1 medium for 30′. Plasmids used were: pcDNA-PTPN22-FLAG, pcDNA-PTPN22R620W (a gift from Dr. Eric Peterson, University of MN, Minneapolis, MN^29^), pSLX-Csk (a gift from Dr. David Schlaepfer, Scripps Research Institute, La Jolla, CA^30^), pcDNA-TRAF3-HA, and pcDNA-TRAF3ΔTRAFC-HA^31^. For each transfection, 8 μg of plasmid and 12.5 μl of lipofectamine 2000 were suspended in Optimem-1 medium, as specified in the company’s protocol, and added to the HEK 293T cells. 6–8 h later medium was removed and fresh DMEM10 was added. Two days later cells were washed with PBS and lysed with 1 × RIPA buffer (150 mM NaCl, 5 mM EDTA [pH 8.0], 50 mM TRIS [pH 8.0], 1% NP-40, 0.5% sodium deoxycholate, 0.1% SDS, 2 mM Na_3_VO_4_, EDTA free mini-complete protease inhibitor tablets, 5 μg/ml DNase 1) for 30′ on ice. Cell lysate was centrifuged for 10′ at 15,000 rpm. The supernatant was saved and an immunoprecipitation was performed as described above using 5 μl of mouse anti-Csk mAb.

### Western blot analysis

Abs used for Western blotting analysis were: mouse anti-Csk (BD Biosciences), rabbit anti-Csk (C-20), rabbit anti-TRAF3 (H122,), mouse anti-CD3_ζ_ (6B10.2, all from Santa Cruz), rabbit anti-pCsk S364 (Abcam), rabbit anti-PTPN22 (D6D1H), rabbit anti-pSrc 416, rabbit anti-Lck, rabbit anti-pLck 505, rabbit anti-LAT (all from Cell Signaling), mouse anti-FLAG (M2), mouse anti-HA (HA-7, all from Sigma), mouse anti-pY (4G10) and mouse anti-actin (Clone 4, Millipore). Western blot analysis was performed as in ref. 13.

### Total internal reflection fluorescence (TIRF) microscopy

Imaging was accomplished using the Leica AM TIRF MC imaging system as in ref. 24 with modifications below. 5 × 10^5^ primary mouse splenic T cells from LMC or T-*traf3* ^−/−^ mice were placed into glass chamber slides (LabTek II) pre-coated with 10 μg/ml anti-CD3 and anti-CD28 Abs. T cells were stimulated for the indicated times. Cells were blocked with SEA blocking buffer (Thermo Scientific) for 1 h and stained with 5 μl rabbit anti-PTPN22 Ab (Proteintech) overnight at 4 °C. Cells were washed and incubated at room temperature with Alexa 488-conjugated goat anti-rabbit IgG (Invitrogen) secondary Ab for 2 h. Cells were washed and fresh PBS was added to each well. Images were obtained using a 100x oil submersion lens and Leica AF software. In individual experiments, all images were obtained using the same laser intensity and exposure parameters. Images were processed using Adobe PhotoShop and ImageJ software. Analysis via ImageJ was performed by circling individual cells and maximum intensity was obtained using colocalization-CoLoc2. Data from approximately 50 cells analyzed/time point were compiled and graphed using scattered dot plots with mean 95% confidence interval (GraphPad Prism 6). Data were obtained from two independent experiments. Statistics were established by one-way Anova with the level of significance p < 0.001 represented as ****. Full-sized, uncropped gels for each Western blot are presented in Suppl. Fig. 5.

## Electronic supplementary material

Supplementary Figures and Legends
