# Supplementary material for: TRAF3 enhances TCR signaling by regulating the inhibitors Csk and PTPN22
Source: Sci Rep. 2017 May 18;7:2081. doi: 10.1038/s41598-017-02280-4 (PMC5437045; doi:10.1038/s41598-017-02280-4)

TRAF3 enhances TCR signaling by regulating the inhibitors Csk and PTPN22

Alicia M. Wallis^1^, Ellie C. Wallace^2^, Bruce S. Hostager^3^, Zuoan Yi^3^, Jon C.D. Houtman^1,3,4^ & Gail A. Bishop^*^.^1,2,3,4,5^

Graduate Program in Immunology^1^, Biomedical Engineering^2^, Depts of Microbiology^3^, Internal Medicine^4^, The University of Iowa and VAMC^5^, Iowa City IA 52242

**Supplemental Material**

**Supplementary Fig. S1. TCR signaling protein levels in TRAF3 deficient T cells.** Protein levels of TRAF3 **(a, c and e)** or TCR signaling proteins **(b and d)** were determined by Western blot analysis in shLUC and shTRAF3 **(a and b)**, HuT28.11 and crTRAF3^-/-^ (Clone 45) **(c and d)** and LMC and T-*traf3*^-/-^ **(e)** whole cell lysates. Western blots were cropped to focus upon specific proteins indicated. Full-length blots are presented in Supplementary Figure S5. Quantification was performed by normalizing relative amounts of indicated proteins to actin and subsequently calculating the fold change of normalized shTRAF3, crTRAF3^-/-^ or T-*traf3*^-/-^ values to the normalized control shLUC, HuT28.11 or LMC values, respectively. Data from at least 3 independent experiments were pooled and the mean values + SEM are shown. Statistical analysis was performed using the Wilcoxon matched-pairs signed rank test, which indicated no statistical differences between TRAF3 deficient vs. sufficient T cells in b and d.

**Supplementary Fig. S2. Loss of TRAF3 decreases CD3_ζ_ activation.** crTRAF3^-/-^ (clone 45) human T cells were stimulated as described in Fig. 1. **(a)** Immunoprecipitation of the CD3/CD28 complex was performed on the whole cell lysates. Blotting for pY and CD3_ζ_, the relative amount of activated and total CD3_ζ_ was analyzed, respectively, by Western blot (top). Data are representative of 2 individual experiments. **(b)** Western blot analysis was performed on whole cell lysates to detect tyrosine phosphorylated proteins and actin. Data from at least 3 independent experiments were pooled. Blots were cropped to focus upon the specific proteins indicated. Full-length blots are presented in Supplementary Figure S5.

**Supplementary Fig. S3. TCR signaling in crTRAF3^-/-^ T cells.** T cells were stimulated via CD3/CD28 for indicated times. Whole cell lysates were prepared from HuT28.11 subclones crTRAF3^-/-^ 28 and 45 **(a, top)** or clone 45 only **(b, top)**, as described in Methods. Western blot analysis was performed to detect the indicated proteins. Western blots were cropped to show proteins indicated. Expression levels of pFyn^Y417^/pLck^Y394^, detected by anti-pSrc^Y416^ Ab, were first normalized to actin as an internal control **(a, bottom)**. Normalization in **(b)** followed as indicated previously; relative levels were further normalized by dividing normalized pSrc protein levels by normalized total Lck levels. Fold change was calculated from the control 0 time point **(a and b, bottom)**. Full-length blots are presented in Supplementary Figure S5. Data from at least 3 independent experiments were pooled and the mean values + SEM are shown. A 2-way ANAOVA was performed to establish statistical significance; * = P<0.05, ** = P<0.01.

**Supplementary Fig. S4. TRAF3 enhances PTPN22 and Csk association in resting T cells.**

T cells were stimulated for 5 minutes via CD3/CD28. An immunoprecipitation for Csk was performed as described in the Methods, using crTRAF3^-/-^ (clone 45) T cell whole cell lysates. Western blots were cropped to focus upon specific proteins indicated. Full-length blots are presented in Supplementary Figure S5. C=Control samples, cells were unstimulated and no immunoprecipitation Ab was added, to detect any nonspecific binding to the protein G beads. Error bars indicate mean values+ SEM of two experiments.

**Supplementary Fig. S5. Uncropped pictures of western blots.** Western blots from figures 1-5 and supplementary figures 1-4 are provided in full-length.


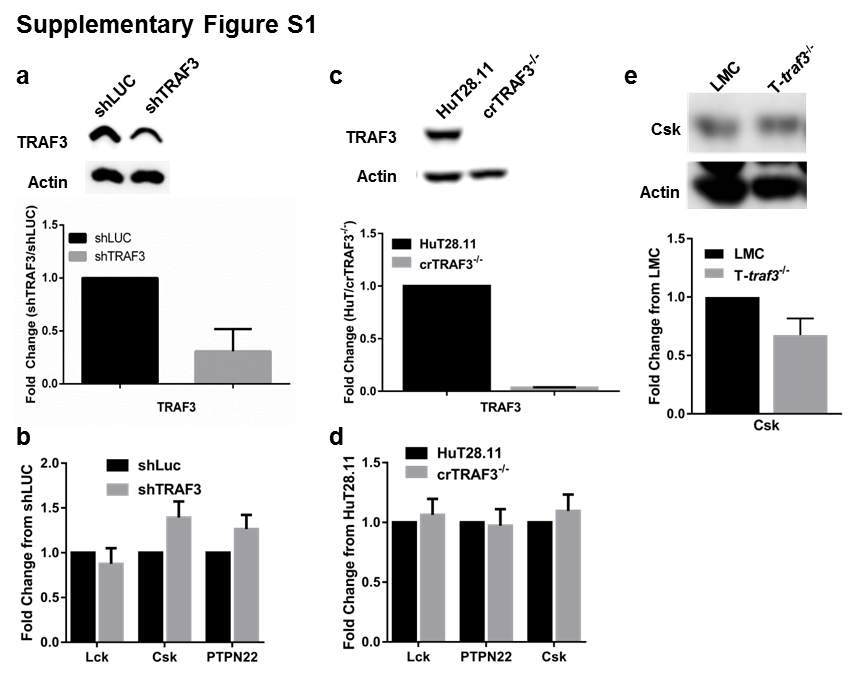


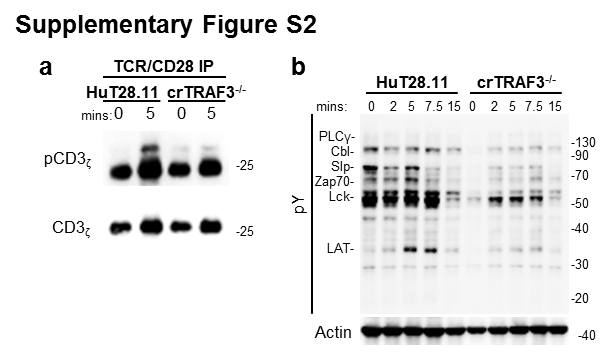


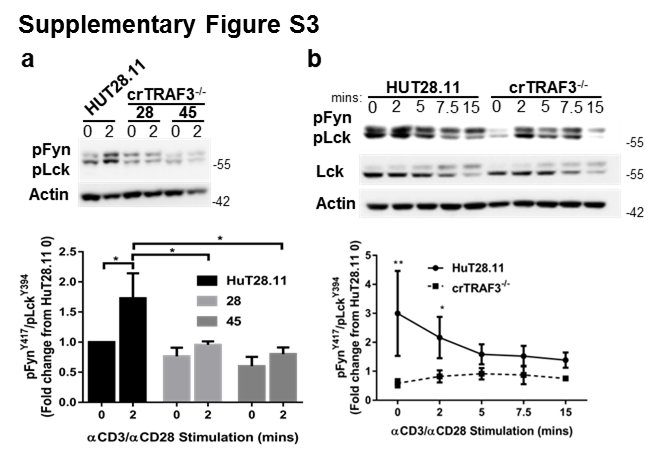


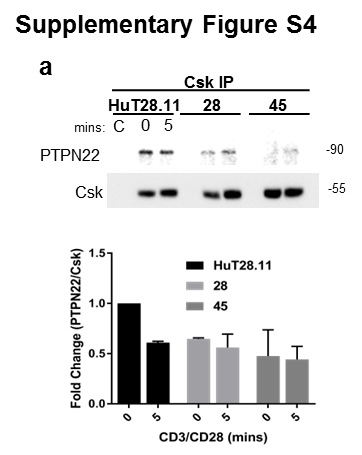


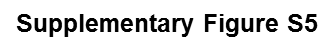

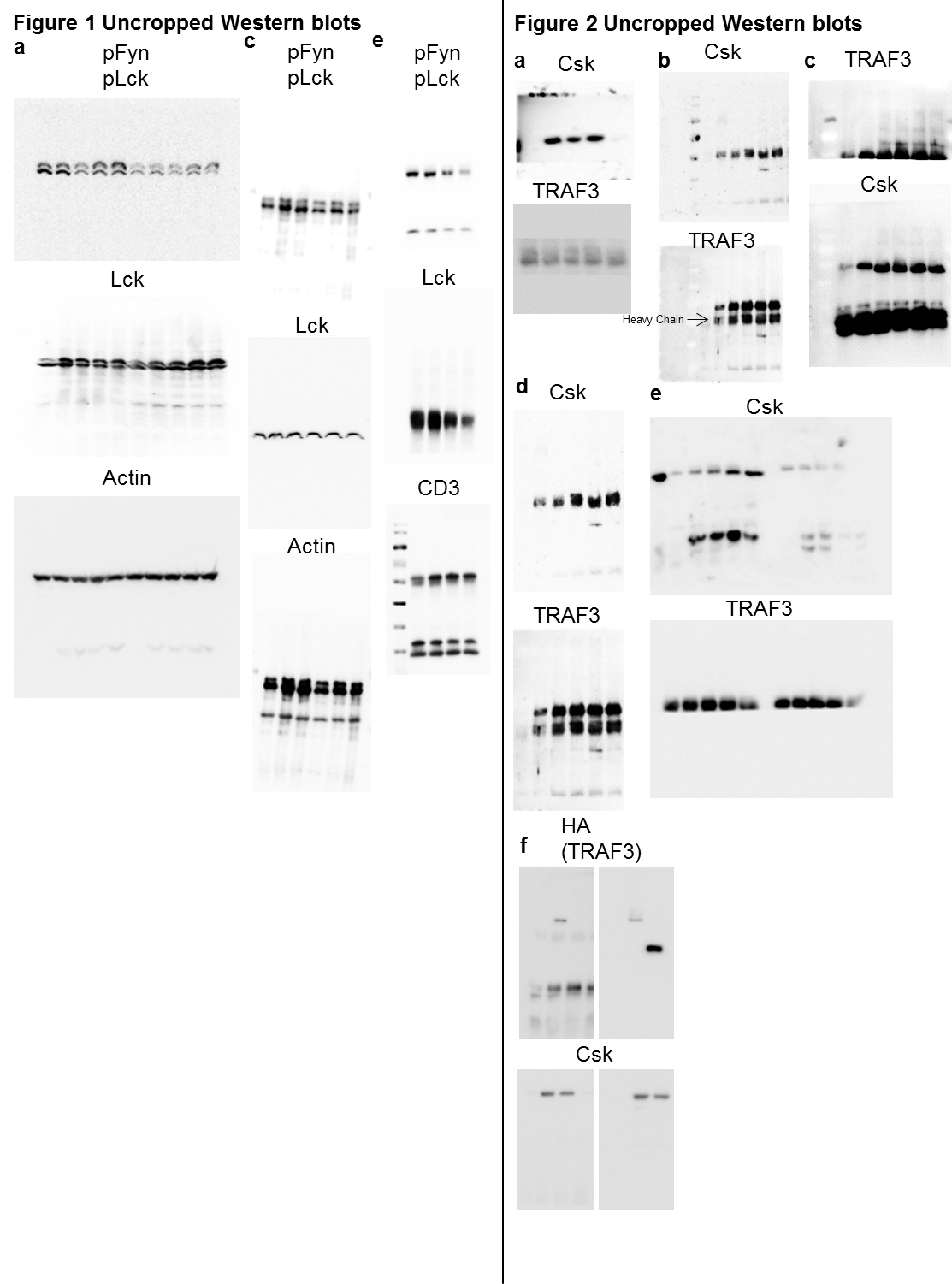

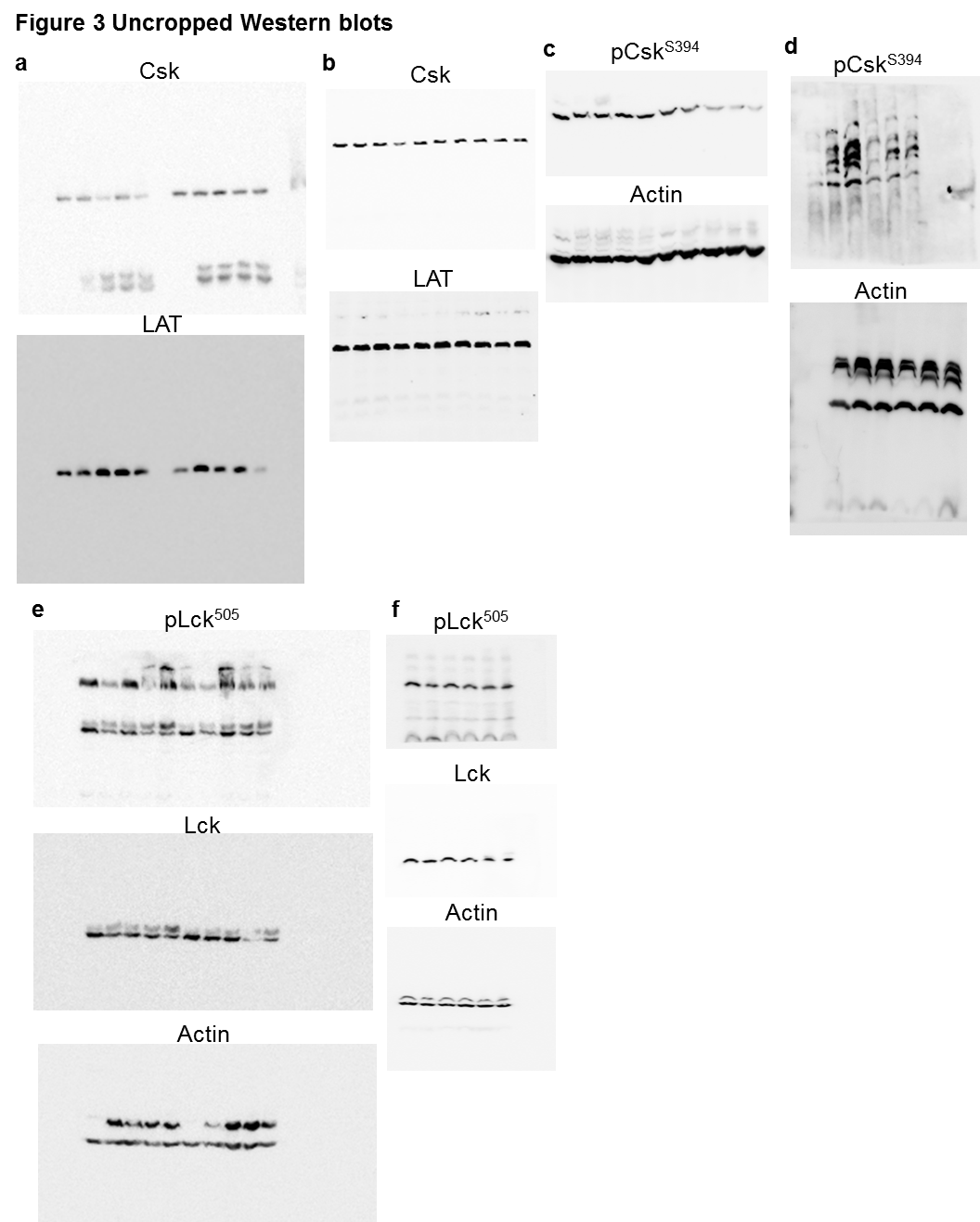

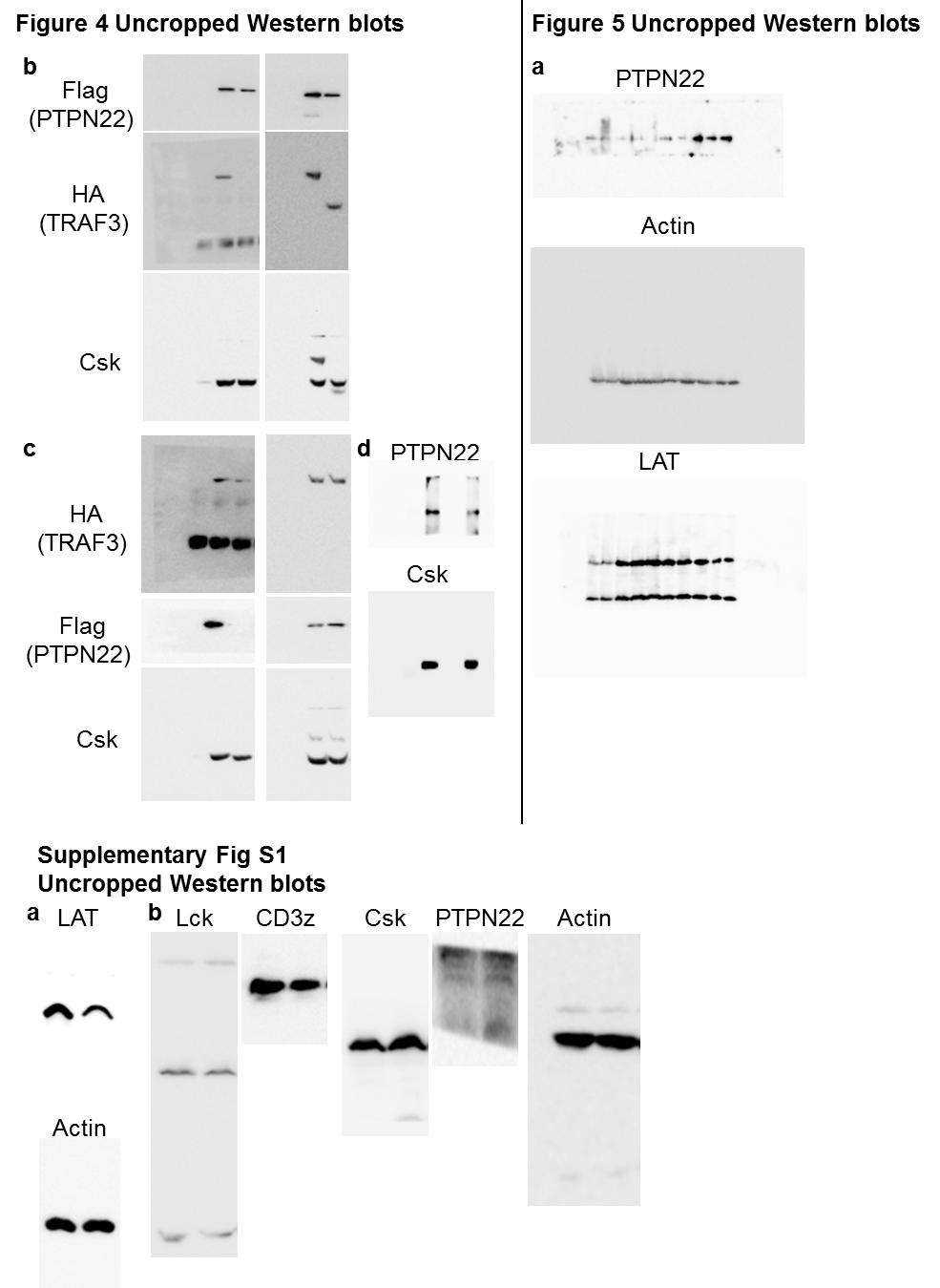


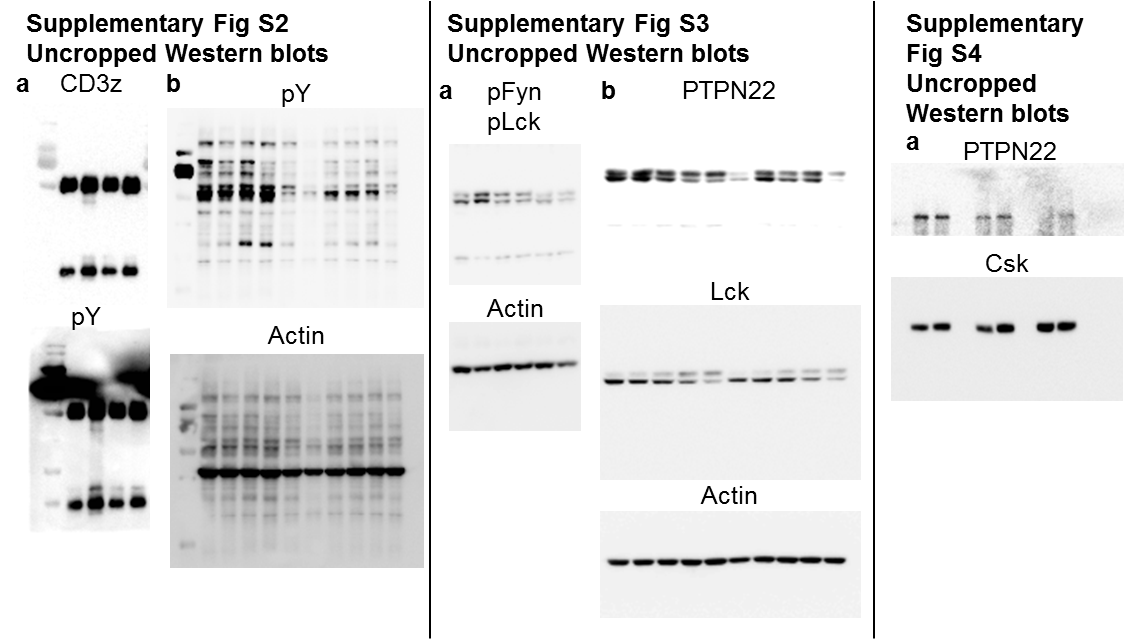

Supplement: Supplementary file 1 — Supplementary Figures and Legends [file 41598_2017_2280_MOESM1_ESM.doc]
